# Intravenous Injection of GluR2-3Y Inhibits Repeated Morphine-Primed Reinstatement of Drug Seeking in Rats

**DOI:** 10.3390/brainsci13040590

**Published:** 2023-03-31

**Authors:** Jianjun Zhang, Zhuo Liu, Xiaodong Liu, Xiaoqian Wang, Longchuan Yu

**Affiliations:** 1 College of Basic Medical, Shanxi University of Chinese Medicine, Jinzhong 030619, China; 2 Shanxi Key Laboratory of Chinese Medicine Encephalopathy, Jinzhong 030619, China; 3 CAS Key Laboratory of Mental Health, Institute of Psychology, Beijing 100101, China; 4 School of Crime Investigation, People’s Public Security University of China, Beijing 100038, China; 5 Beijing Institute of Chinese Medicine, Beijing University of Chinese Medicine, Beijing 100029, China; 6 School of Life Sciences, Peking University, Beijing 100871, China

**Keywords:** addiction, GluR2-3Y, long-term depression, morphine, relapse, self-administration

## Abstract

Studies have demonstrated that the α-amino-3-hydroxy-5-methylisoxazole-4-propionic acid (AMPA) receptor is essential to drug addiction. In this study, we explored the influence of GluR2-3Y, an interfering peptide to prevent the endocytosis of AMPA receptors containing the GluR2 subunit, on morphine-seeking behavior in the rat self-administration model. After self-administration was established, the rats received intravenous injections of GluR2-3Y during the extinction sessions. There were no significant differences in both active and inactive pokes compared to the control group of rats that received GluR2-3S, indicating that GluR2-3Y has no significant influences on the extinction of morphine self-administration. The other two groups of rats were trained, extinguished, and reinstated by repeated morphine priming (respectively, called Prime 1, Prime 2, and Prime 3). Only one intravenous injection of GluR2-3Y was performed before Prime 1. Compared to the control group, GluR2-3Y did not affect Prime 1, but significantly attenuated the morphine-seeking behavior during repeated morphine-primed reinstatement, indicating an inhibitory after effect of GluR2-3Y on morphine-seeking behavior in rats. The long-term depression (LTD) in the nucleus accumbens (NAc) shell was also assessed. Pretreatment with GluR2-3Y altered the ability of LTD induction to the level of that in the naive group, while pretreatment with GluR2-3S had no effects on LTD. Our results demonstrated that the intravenous injection of GluR2-3Y, to block the endocytosis of AMPA receptors, inhibited the reinstatement of morphine-seeking behavior, which may be induced by modulating the neuronal plasticity in the NAc shell of rats.

## 1. Introduction

Addiction is thought to be a chronically relapsing disease/disorder. The process of drug addiction exploits the reward-related learning and memory systems [1] and involves mechanisms of synaptic plasticity [2,3,4]. For instance, exposure to different drugs of abuse induces changes in long-term potentiation (LTP) or long-term depression (LTD), two main forms of Hebbian synaptic plasticity, in the neural circuits underlying reward-related learning such as the nucleus accumbens (NAc) [5,6] and hippocampus [7]. Meanwhile, altering the synaptic plasticity in these brain regions also influences addiction behavior [8,9,10].

It is well known that different types of glutamate receptors play important and specific roles in synaptic transmission and plasticity [11,12,13,14,15,16,17,18,19,20,21]. The AMPA receptor, one of the three types of ionotropic glutamate receptors (iGluRs), is integral to synaptic plasticity [22,23,24] and is involved in opioid addiction [25,26,27,28,29]. For example, after a single morphine injection, the mRNA levels of AMPA receptor subunits were significantly reduced three days later but enhanced three weeks later in the NAc core [30]. The AMPA receptor GluR1 subunit on the dendrite membrane in the basolateral amygdala (BLA) increased in morphine self-administering rats [31]. The surface expression of the AMPA receptors changed in the medial prefrontal cortex (mPFC) upon repeated morphine administration [32]. Acute or chronic morphine administration produced region-specific changes in the subcellular distribution of the GluR1 subunit in the rat ventral tegmental area (VTA) [33]. Overexpression of the GluR1 subunits or knockdown of the GluR2 subunits in the central amygdala (CeA) facilitated the acquisition of morphine-induced conditioned place preference (CPP) [34].

Among the subunits of the AMPA receptor, GluR2 regulates synaptic plasticity by controlling Ca^2+^ influx [35,36]. The presence of the GluR2 subunit in the heteromeric AMPA receptor is Ca^2+^-impermeable. An early study by Van den Oever and colleagues found that preventing GluR2 endocytosis decreased cue-induced heroin-seeking behavior [37]. However, its specific role in relapse to opioids is still not clear, especially the synaptic changes that take place after morphine priming, which may lead to the resumption of drug seeking, have not yet been studied.

GluR2-3Y is a GluR2-derived interfering peptide that contains tyrosine residues and acts as a competitive inhibitor of tyrosine phosphorylation. The accumulated evidence shows that GluR2-3Y blocks the endocytosis of the AMPA receptors containing GluR2 and the induction of LTD [38,39,40,41]. Besides, many studies have demonstrated that GluR2-3Y influences the process of learning and memory and addictive behavior induced by different drugs of abuse. For example, both the systemic and intra-NAc infusion of GluR2-3Y prevented the expression of amphetamine-induced behavioral sensitization in the rat [39]. Pretreatment of GluR2-3Y inhibited the acquisition and reinstatement of morphine CPP in rats [42], indicating the AMPA receptor endocytosis is a target for the treatment of opioid addiction and GluR2-3Y is of great value in the treatment.

Therefore, in the present study, using GluR2-3Y, we explored the effect of inhibiting AMPA receptor endocytosis on the reinstatement of drug-seeking behavior induced by morphine priming in a repeated priming model. We also studied the involvement of LTD induced in the NAc shell in the process.

## 2. Materials and Methods

### 2.1. Animals 

Male Sprague–Dawley rats (220–250 g) were afforded one week of acclimation before use. The rats were housed under a reverse cycle (12 h light/dark) with food and water ad libitum, except for the morphine self-administration (SA) training when they received a 20 g ration of rat chow daily.

### 2.2. Drugs

Morphine hydrochloride injection (Shenyang First Pharmaceutical Factory, Shenyang, China) was diluted with sterile saline to obtain a 1 mg/mL dose. GluR2-3Y (YGRKKRRQRRR-YKEGYNVYG) and scrambled control peptide GluR2-3S (YGRKKRRQRRR-VYKYGGYNE) (GL Biochem, Shanghai, China) were dissolved in sterile saline.

### 2.3. Surgery

Under sodium pentobarbital anesthesia (75 mg/kg, i.p.), a silastic catheter was implanted in the right jugular vein. After surgery, the catheter was flushed with heparinized saline (100 IU/mL, 0.4 mL) daily. The rats were allowed to recover for 7 to 10 days after surgery.

### 2.4. Morphine SA, Extinction, and Relapse Tests

#### 2.4.1. Apparatus

The SA setup consisted of 16 operant chambers (29 × 29 × 26 cm; AniLab, Ningbo, China). Each chamber was located in a sound-proof opaque box equipped with exhaust fans and which had a white house light for illumination. There were two holes, and a blue cue light was placed inside each hole. A speaker was used to provide audio cues. Each rat was placed in a chamber daily, and the catheter was connected to a pump-driven syringe. The data were collected with AniLab software (AniLab, Ningbo, China).

#### 2.4.2. Acquisition

The rats were trained to perform morphine self-administration (0.3 mg/kg/100-µL injection) on a fixed ratio 1 (FR1) schedule in daily 3 h sessions during their dark cycle. The house light was turned on at the beginning of each session and indicated the availability of morphine. Poking the nose into the active hole caused an infusion of morphine (5 s) and simultaneously caused a 5 s compound audiovisual cue, which meant that the blue light placed inside the hole lit up and the speaker sounded. Poking the nose into the inactive hole had no consequences. The injection of morphine was followed by a 15 s time out, during which time the house light was turned off, and the nose pokes were ineffective.

#### 2.4.3. Extinction

Extinction sessions were conducted for three hours once daily. The procedures for the extinction sessions were identical to the training sessions, except that morphine was not available.

#### 2.4.4. Relapse

On the test day, the rats were injected with 5 mg/kg of morphine intraperitoneally and immediately placed in the operant cages. The procedures for the relapse session were the same as the extinction session.

#### 2.4.5. Behavior Experiment 1: Effect of Intravenous Pretreatment with GluR2-3Y on the Extinction of Morphine SA

The rats were trained to self-administer morphine for 14 days, followed by 21 consecutive extinction sessions. The 14 rats were administered with GluR2-3Y (n = 7) or GluR2-3S (n = 7) (1.5 nmol/g body weight, i.v.) 60 min before the extinction sessions for the first 19 days.

#### 2.4.6. Behavior Experiment 2: Effect of Intravenous Pretreatment with GluR2-3Y on the Morphine-Primed Reinstatement of Drug Seeking

Another two groups of rats were used. The rats remained in morphine SA training until they met an acquisition criterion that required the average infusion over three consecutive training days to vary by less than 15%. Once the rats met this criterion, the extinction procedures were instituted on the following day. The rats remained in extinction until the active pokes and inactive pokes were both less than 10 for three consecutive days. Twenty-four hours after meeting this criterion, 17 rats were reinstated with morphine (5 mg/kg, i.p.) for Prime 1. GluR2-3Y (n = 8) or GluR2-3S (n = 9) (1.5 nmol/g body weight) was intravenously injected 60 min before morphine Prime 1 (a three-hour session). The extinction procedures were instituted for at least three days, and after meeting the extinction criterion, Prime 2 (a three-hour session) was performed 24 h later. Then, the extinction procedures were instituted for at least another three days, and Prime 3 (a three-hour session) was performed after meeting the extinction criterion.

### 2.5. Electrophysiological Studies 

#### 2.5.1. Slice Preparation 

Twenty rats were used. The number of rats in the naive group, GluR2-3Y, and GluR2-3S was eight, six, and six, respectively. Slices were prepared as described previously with minor modifications [43]. One day after meeting the extinction criterion, the rats were anesthetized with sodium pentobarbital (75 mg/kg, i.p.) and decapitated. The brain was quickly removed into an ice-cold cutting solution containing (mM) 87 NaCl, 2.5 KCl, 7 MgCl_2_, 0.5 CaCl_2_, 1.25 NaH_2_PO_4_, 25 NaHCO_3_, 15 glucose, and 90 sucrose and cooled for 3–5 min. Then, 300-μm sagittal slices were cut on a vibratome (Leica VT 1000 S, Heidelberg, Germany) in the ice-cold cutting solution. Immediately after cutting, the slices were stored for 45 min at 33 °C in artificial cerebrospinal fluid (ACSF) containing (mM) 125 NaCl, 2.5 KCl, 1 MgCl_2_, 2 CaCl_2_, 1.25 NaH_2_PO_4_, 25 NaHCO_3,_ and 10 glucose, and were equilibrated with 95% O_2_/5% CO_2_. The slices were then stored at room temperature until recording.

#### 2.5.2. Patch Clamp 

Recordings were conducted in a chamber superfused continuously with carbonated ACSF. The superfusion medium contained bicuculline (Sigma, St. Louis, MO, USA, ten μM) to block the GABAA receptors. Whole-cell recordings were performed from visualized neurons in the NAc shell. Glass microelectrodes (resistance 4–6 MΩ) were filled with a solution containing (mM) 122.5 Cs-gluconate, 17.5 CsCl, 2 MgCl_2_, 10 HEPES, 0.5 EGTA, 4 ATP, and osmolarity 300–310 mOsm. The pH was adjusted to 7.2–7.4 with CsOH. The data were recorded with a Heka EPC10 amplifier (Heka, Reutlingen, Germany).

#### 2.5.3. LTD Recordings 

Synaptic currents were evoked by stimulating the prelimbic cortical synaptic inputs via a constant-voltage pulse (1 ms) delivered through a concentric bipolar electrode (FHC, Bowdoinham, ME, USA). Synaptic responses were evoked at 0.067 Hz, except during the induction of LTD. After 10 min of stable baseline recording, LTD was triggered by pairing low-frequency stimulation (1 Hz, 480 pulses) while holding the cells at −40 mV.

### 2.6. Data Analysis

We analyzed the data with Prism GraphPad (version 8). Data from the SA and electrophysiological recordings were analyzed with one-way or repeated measures analysis of variance (ANOVA) with a Bonferroni post-test. The critical value for statistical significance was set at *p* < 0.05. Data are presented as mean ± SEM.

## 3. Results

### 3.1. Effects of Intravenous GluR2-3Y Injection on the Extinction of Morphine SA

**Figure 1:**
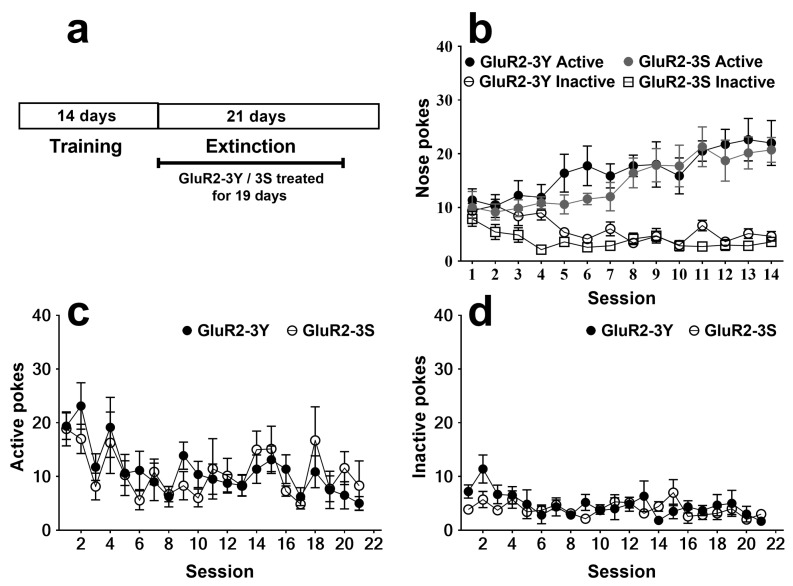
Intravenous injection of GluR2-3Y has no effect on the extinction of morphine SA. (**a**) Experimental protocol. (**b**) Active and inactive pokes during morphine SA training. (**c**) Active pokes during extinction sessions. (**d**) Inactive pokes during extinction sessions. Data are expressed as mean ± s.e.m. (n = 7 per group).

After 14 days of training, the rats that acquired morphine SA were divided into two groups for extinction. One group received intravenous injections of GluR2-3Y (1.5 nmol/g body weight, n = 7), and the other group received intravenous injections of GluR2-3S (1.5 nmol/g body weight, n = 7) as a control. For the 14 days of SA training, a three-way analysis of variance revealed a significant session effect (F 13, 156 = 2.616; *p* < 0.01) and nose poke effect (F 1, 12 = 31.80; *p* < 0.001), with no significant group effect (F 1, 12 = 3.177; *p* = 0.100) or group × nose poke × session interaction (F 13, 156 = 0.877; *p* = 0.579). No significant difference was found for the nose pokes in the two groups (Figure 1b). 

The effects of GluR2-3Y given just before 19 daily extinction sessions are shown in Figure 1c, d. The rats that received the GluR2-3Y treatment showed no significant difference in both active pokes (F 1, 240 = 0.003; *p* > 0.05; Figure 1c) and inactive pokes (F 1, 240 = 1.32; *p* > 0.05; Figure 1d) compared to the rats that received the GluR2-3S injection, indicating that GluR2-3Y had no significant influences on extinction.

### 3.2. Effects of Injection of GluR2-3Y on Morphine-Seeking Behavior during Repeated Morphine-Primed Reinstatement

**Figure 2:**
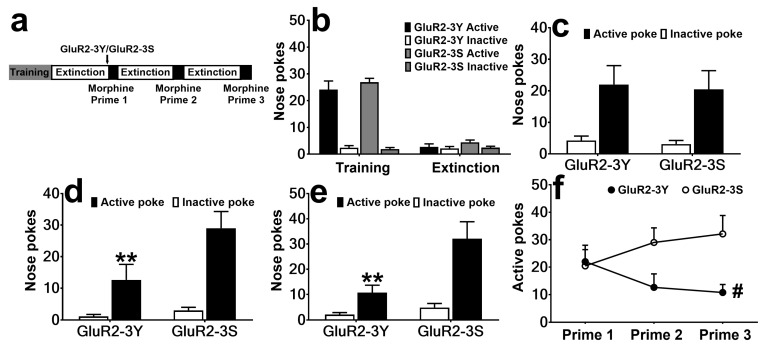
Intravenous injection of GluR2-3Y attenuates morphine-induced morphine-seeking. (**a**) Experimental protocol. (**b**) Active and inactive pokes during morphine SA training and extinction, showing the last day of training and extinction. (**c**–**e**) Nose pokes during the morphine Prime 1, Prime 2, and Prime 3 sessions. (**f**) Active pokes during the three reinstatement sessions. # indicates a significant difference between the GluR2-3Y and GluR2-3S groups, and ** *p* < 0.01 indicates significant differences in the Bonferroni post-test pairwise comparison vs. the active pokes of the GluR2-3S group. Data are expressed as mean ± s.e.m. (n = 8–9 per group).

The rats that met the extinction criterion were divided into two groups with no significant differences for the nose pokes (F 1, 15 = 0.40, *p* > 0.05 for the last training day, F 1, 15 = 1.11, *p* > 0.05 for the last extinction day; Figure 2b). The rats received intravenous injections of GluR2-3Y (n = 8) or GluR2-3S (n = 9), then were reinstated with morphine. As shown in Figure 2c, GluR2-3Y did not affect Prime 1 (F 1, 15 = 0.09, *p* > 0.05). To determine whether there were after effects of GluR2-3Y or not, Prime 2 and Prime 3 progressed, and interestingly, significant attenuation effects were found in both Prime 2 and Prime 3 (F 1, 15 = 5.91, *p* < 0.05 for Prime 2, F 1, 15 = 8.98, *p* < 0.01 for Prime 3; Figure 2d, e). The Bonferroni post-test showed GluR2-3Y significantly attenuated the active pokes (t 15 = 3.14, *p* < 0.01 for Prime 2, t 15 = 3.84, *p* < 0.01 for Prime 3), but not the inactive pokes (t 15 = 0.36, *p* > 0.05 for Prime 2, t 15 = 0.50, *p* > 0.05 for Prime 3). Combining the data from the three reinstatement tests, we found GluR2-3Y attenuated morphine-primed morphine-seeking behavior during repeated morphine-primed reinstatement (F 1, 15 = 4.71, *p* < 0.05). 

### 3.3. Influences of GluR2-3Y on the LTD in NAc Shell Neurons before Morphine-Primed Reinstatement

**Figure 3:**
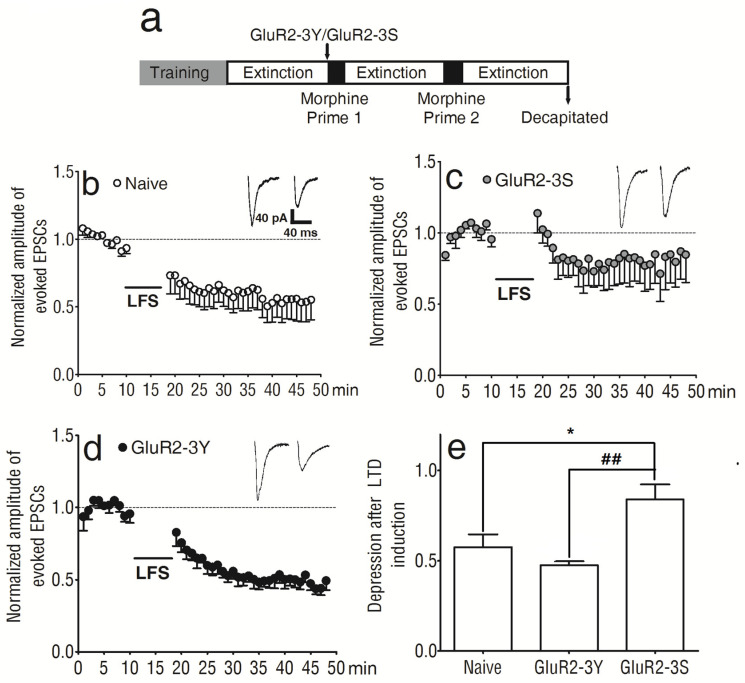
Intravenous injection of GluR2-3Y altered LTD in the NAc shell. (**a**) Experimental protocol. (**b**) Expression of LTD in the NAc of naive rats. (**c**) Expression of LTD in the NAc of animals treated with GluR2-3S. (**d**) Expression of LTD in the NAc of animals treated with GluR2-3Y. (**e**) Bar graphs summarizing the data for the last 5 min of LTD induction from experiments such as those shown in (**b**–**d**). ## *p* < 0.01 versus the GluR2-3S group, * *p* < 0.05 versus the naive group (Bonferroni post-test). Data are expressed as mean ± s.e.m. (n = 6–8 per group).

To determine the involvement of synaptic plasticity underlying the attenuating effect of GluR2-3Y on repeated morphine-primed reinstatement, we explored the changes in LTD induced in the NAc shell. Another group of rats was used, and GluR2-3Y and GluR2-3S were injected as before. After reaching the extinction criterion, the rats were anesthetized and decapitated for brain slice preparation (Figure 3a).

LTD induction was recorded in the NAc shell of naive rats (Figure 3b), as well as those pretreated with GluR2-3S (Figure 3c) or GluR2-3Y (Figure 3d). All the data were normalized to the average value of the evoked EPSCs before LTD induction. The last 5 min of LTD induction showed significant differences between the 3 groups (F 2, 99 = 7.31, *p* < 0.01; Figure 3e); there were significant differences between the naive and GluR2-3S-pretreated rats (t99 = 2.86, *p* < 0.05) and between the GluR2-3Y- and GluR2-3S-pretreated rats (t99 = 3.68, *p* < 0.01; Figure 3e). The amplitude of the normalized evoked EPSCs was 57.44 ± 7.09% in the naive rats (n = 8), 47.53 ± 2.17% in the GluR2-3Y-treated rats (n = 6), and 83.88 ± 8.29% in the GluR2-3S-treated rats (n = 6). The data indicated that GluR2-3Y attenuated the morphine-primed morphine-seeking behavior. This inhibitory effect may be associated with its influence on the synaptic plasticity in the NAc shell, but GluR2-3S did not have the same effect.

## 4. Discussion

Morphine and other opioid drugs are widely used to treat pain, but their clinical usefulness is limited by tolerance and dependence [44]. To develop effective therapies for morphine addiction, it is crucial to prevent relapse. The drug, the cue associated with the drug of abuse, and stress all induce relapse [45,46,47]. Here, we showed that inhibiting the endocytosis of AMPA receptors with the interfering peptide GluR2-3Y attenuated the reinstatement of morphine-seeking behavior induced by repeated morphine-priming in rats trained to morphine SA, but had no effect on extinction. Furthermore, we found that the LTD induction in the NAc shell of rats returns to the normal level as the naive rats as a result of pretreatment with GluR2-3Y. So, the rescue of LTD in the NAc shell might contribute to the reduction in morphine-seeking behavior. The data demonstrated that GluR2-3Y is a potential candidate for treating opioid addiction, especially the prevention of relapse.

Previous studies have shown that drugs of abuse influence the glutamate system and synaptic plasticity [22,23,48]. For instance, cocaine changes the release of glutamate in several brain areas [49,50], and exposure to cocaine or morphine alters the expression of the AMPA receptors in the prefrontal cortex (PFC) [32], the VTA [33], and the NAc [51,52,53]. For opioid addiction, reinstatement by either heroin or cue increases extracellular glutamate in the NAc core in the self-administration rats [54]. Moreover, chronic morphine administration induces a decrease in the surface AMPA receptor GluR1 subunit in particular types of neurons in rats’ NAc shell and NAc core [55]. However, our results suggested that the endocytosis of GluR2 was not involved in the extinction of morphine self-administration, consistent with the previous finding on morphine-conditioned place preference (mCPP) [25].

Besides, an earlier study showed that GluR2-3Y decreases cue-induced heroin reinstatement [37]. Here, we showed that the infusion of GluR2-3Y reduced the reinstatement in a delayed and persistent manner in our repeated priming model, which suggests the possible effect of GluR2 endocytosis on morphine-induced relapse. However, inconsistent with the effect of GluR2-3Y on cue-induced heroin reinstatement, morphine Prime 1 was not affected. These differences may be due to differences in the ways of induction. One previous study demonstrated that low-dose risperidone can attenuate cue-induced but not heroin-induced reinstatement [56]. Similarly, another study showed that ketanserin can attenuate cue-induced but not cocaine-induced reinstatement [57]. These studies suggested that cue-induced and drug-induced reinstatement have different mechanisms, which need to continue to be explored.

The reasons why Prime 2 and Prime 3 were affected by intravenous injections of GluR2-3Y may be explained by the widely accepted hypothesis that addiction is a pathological process that involves plasticity mechanisms related to the glutamate system, similar to those implicated in neuronal models of learning and memory [58,59,60,61]. When exposed to the drug use-related cue/context or the drug itself, the memories of the drug experience are retrieved, leading to drug seeking. During the reinstatement induced by morphine priming, a small dose of morphine triggers the drug-seeking behavior, which might be due to the activation of the association between drug-use experience and drug-seeking behavior. While established memory will be labile and, then, sensitive to disruption [62]. Milekic et al. found that established morphine-conditioned place preference was persistently disrupted if protein synthesis was blocked after the re-presentation of a conditioning session, and this effect required the re-experience of both the conditioning context and the drug [63]. Rao-Ruiz et al. found that contextual fear memory recall induced acute hippocampal AMPAR endocytosis [64], suggesting that GluR2 plays a crucial role in memory reconsolidation. So, the finding that GluR2-3Y injection attenuated morphine-seeking behavior during repeated morphine-primed reinstatement sessions may be due to GluR2-3Y interfering with the memory reconsolidation after the first morphine-primed reinstatement. The effects of intravenous GluR2-3Y injection on the extinction after Prime 1 and Prime 2 also support this supposition (Figure S1). The time course and the relationship between reinstatement and memory reconsolidation need more research.

Previous studies on drug addiction underscore the importance of the NAc in the development of drug seeking, extinction, and relapse [65,66,67]. The NAc shell, an area that processes reward-related incentives [55], is critically involved in the reward effect [68,69]. Moreover, the enhancement of control over behavior by psychostimulants depends on the shell [70,71]. Although LaLumiere and colleagues indicated that the glutamate receptors in the NAc core play an essential role in heroin reinstatement [54], Peters and colleagues also identified that inhibition of the shell induced cocaine-seeking in extinguished rats [72]. Here, we demonstrated the critical function of the NAc shell in morphine-induced reinstatement. In addition, memory reconsolidation seems to be involved in neuroplasticity [73]. Other evidence indicates the role of AMPARs endocytosis and LTD in behavioral sensitization [39,74]. Therefore, we hypothesized that the plasticity of the NAc shell was related to the activation of an association between morphine-use experience and morphine-seeking behavior, and our observations fit within this conceptual framework.

There are several limitations to this study. Firstly, we only tested male rats; thus, how GluR2-3Y may be involved in the priming of morphine SA in female animals is not known. Secondly, we did not record the other behavioral parameters, such as the latency to poke, and the timeline of poking among the length of the session; if GluR2-3Y affects these parameters needs to be further investigated.

## 5. Conclusions

Our results showed that relapse induced by morphine priming can be persistently disrupted by the intravenous injection of GluR2-3Y. This effect may be due to a change of LTD in the NAc shell. This finding suggests that GluR2-3Y influenced the synaptic plasticity in the NAc shell and interfered with the morphine-induced reconsolidation of memories related to morphine SA, therefore inhibiting the late reinstatement of morphine-seeking behavior.

## Data Availability

The data presented in this study are available on request from the corresponding author.

## Supplementary Materials

The following supporting information can be downloaded at: https://www.mdpi.com/article/10.3390/brainsci13040590/s1, Figure S1: Effects of intravenous GluR2-3Y injection on extinction after Prime.
