# Supplementary material for: Intravenous Injection of GluR2-3Y Inhibits Repeated Morphine-Primed Reinstatement of Drug Seeking in Rats
Source: Brain Sci. 2023 Mar 31;13(4):590. doi: 10.3390/brainsci13040590 (PMC10136422; doi:10.3390/brainsci13040590)
Supplement: Supplementary file 1 [file brainsci-13-00590-s001.zip › brainsci-2276900-supplementary.pdf]

Supplemental information:

Supplemental Results

Effects of intravenous GluR2-3Y injection on extinction after Prime

After Prime 1 and Prime 2, extinction procedures were instituted for at least three days. During the extinction after Prime 1, three way analysis of variance did not reveal significant session effect ($F_{2, 30} = 1.035$; $p = 0.368$) or nose poke effect ($F_{1, 15} = 3.992$; $p = 0.064$), with no significant group effect ($F_{1, 15} = 3.731$; $p = 0.073$) or group × nose poke × session interaction ($F_{2, 30} = 0.960$; $p = 0.394$). Thus, No significant difference was found for the nose pokes in the two groups (Figure S1a). During the extinction after Prime 2, three way analysis of variance revealed significant group effect ($F_{1, 15} = 5.982$; $p < 0.05$) and nose poke effect ($F_{1, 15} = 6.278$; $p < 0.05$), with no significant session effect ($F_{2, 30} = 1.060$; $p = 0.359$) or group × nose poke × session interaction ($F_{2, 30} = 0.344$; $p = 0.712$). This significant difference found for the nose pokes in the two groups (Figure S1b) was consistant with the difference in Prime 2.

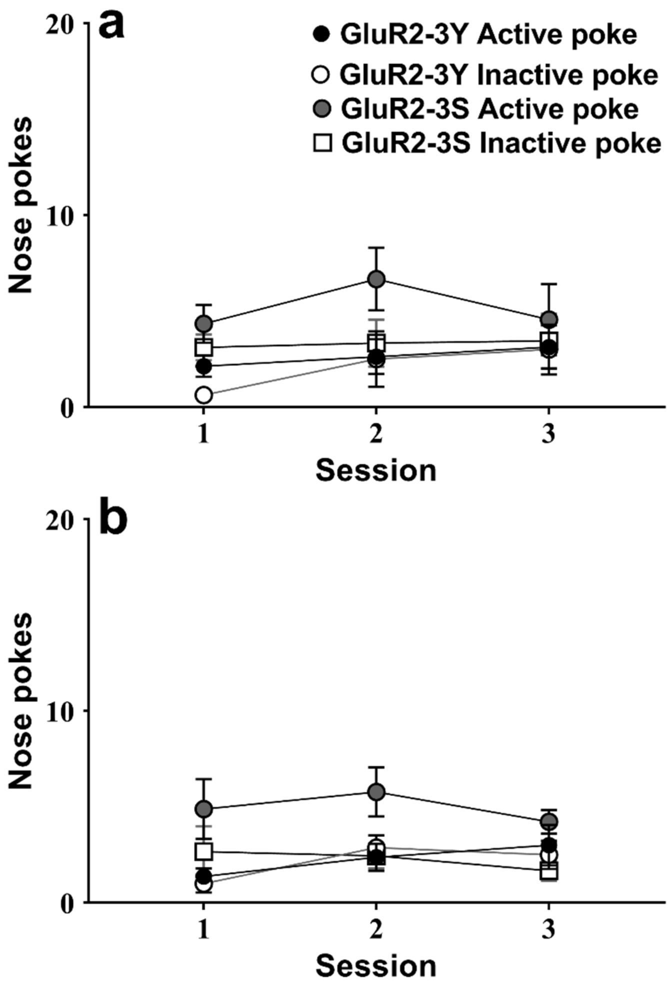

Figure S1. Effects of intravenous GluR2-3Y injection on extinction after Prime.

(a) Active and inactive pokes during the first three days of extinction after Prime 1. (b) Active and inactive pokes during the first three days of extinction after Prime 2. Data are expressed as mean ± s.e.m.
